# The time-resolved atomic, molecular and optical science instrument at the Linac Coherent Light Source

**DOI:** 10.1107/S1600577522004283

**Published:** 2022-05-19

**Authors:** Peter Walter, Timur Osipov, Ming-Fu Lin, James Cryan, Taran Driver, Andrei Kamalov, Agostino Marinelli, Joe Robinson, Matthew H. Seaberg, Thomas J. A. Wolf, Jeff Aldrich, Nolan Brown, Elio G. Champenois, Xinxin Cheng, Daniele Cocco, Alan Conder, Ivan Curiel, Adam Egger, James M. Glownia, Philip Heimann, Michael Holmes, Tyler Johnson, Lance Lee, Xiang Li, Stefan Moeller, Daniel S. Morton, May Ling Ng, Kayla Ninh, Jordan T. O’Neal, Razib Obaid, Allen Pai, William Schlotter, Jackson Shepard, Niranjan Shivaram, Peter Stefan, Xiong Van, Anna Li Wang, Hengzi Wang, Jing Yin, Sameen Yunus, David Fritz, Justin James, Jean-Charles Castagna

**Affiliations:** a SLAC National Accelerator Laboratory, 2575 Sand Hill Road, Menlo Park, CA 94025, USA; b Stanford PULSE Institute, SLAC National Accelerator Laboratory, 2575 Sand Hill Road, Menlo Park, CA 94025, USA

**Keywords:** LCLS-II, FEL, AMO, attosecond

## Abstract

The newly constructed time-resolved atomic, molecular and optical science instrument (TMO) is configured to take full advantage of both linear accelerators at SLAC National Accelerator Laboratory, the copper accelerator operating at a repetition rate of 120 Hz providing high per-pulse energy as well as the superconducting accelerator operating at a repetition rate of about 1 MHz providing high average intensity. Both accelerators power a soft X-ray free-electron laser with the new variable-gap undulator section. With this flexible light source, TMO supports many experimental techniques not previously available at LCLS and will have two X-ray beam focus spots in line. Thereby, TMO supports atomic, molecular and optical, strong-field and nonlinear science and will also host a designated new dynamic reaction microscope with a sub-micrometer X-ray focus spot. The flexible instrument design is optimized for studying ultrafast electronic and molecular phenomena and can take full advantage of the sub-femtosecond soft X-ray pulse generation program.

## Abstract 2

The newly constructed time-resolved atomic, molecular and optical science instrument (TMO), configured to take full advantage of both linear accelerators at SLAC National Accelerator Laboratory, the copper accelerator operating at a repetition rate of 120 Hz providing high per-pulse energy as well as the superconducting accelerator operating at a repetition rate of about 1 MHz providing high average intensity, is described.

## 1. Introduction

**Figure 1:**
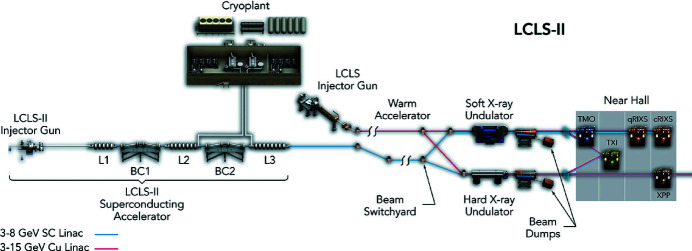
Schematic overview of the new LCLS and NEH layout with both accelerators [LCLS (red) and LCLS-II (blue)]. The LCLS-II X-ray laser is shown alongside the existing LCLS. LCLS uses the last third of SLAC’s two-mile-long linear accelerator – a hollow copper structure that operates at room temperature and allows the generation of 120 X-ray pulses per second. For LCLS-II, the first third of the copper accelerator has been replaced with a superconducting one, capable of a repetition rate of up to 1 MHz. Future Far Hall layout changes for LCLS-II HE are not included.

The unique capabilities of LCLS (Arthur *et al.*, 2002 ▸; Emma *et al.*, 2010 ▸), the world’s first hard X-ray free-electron laser (FEL), have had significant impact on advancing our understanding across a broad range of scientific fields, from fundamental atomic and molecular physics to condensed matter, catalysis, and structural biology (Young *et al.*, 2010 ▸; Seibert *et al.*, 2011 ▸; Chapman *et al.*, 2011 ▸; Gomez *et al.*, 2014 ▸; Bostedt *et al.*, 2016 ▸; Hartmann *et al.*, 2018 ▸; O’Neal *et al.*, 2020 ▸; Ilchen *et al.*, 2021 ▸). A major upgrade of the LCLS facility, the LCLS-II project, is now underway. LCLS-II is being developed as a high-repetition-rate FEL. It features a 4 GeV continuous-wave superconducting linac that is capable of producing uniformly spaced (or programmable) ultrafast X-ray laser pulses at a repetition rate up to 1 MHz spanning the energy range from 0.25 to 5 keV. Fig. 1 ▸ shows a schematic overview of the new FEL layout with both accelerators LCLS and LCLS-II.

To make the performance of the new high-repetition-rate FEL available for three new instruments, the existing near experimental hall (NEH) of LCLS was completely reconfigured and hosts two new soft X-ray instruments: the time-resolved atomic molecular and optical (TMO) instrument and the resonant inelastic X-ray scattering (RIX) instrument, and one tender X-ray instrument, the tender X-ray imaging (TXI) instrument (see Fig. 1 ▸). The first of these instruments, TMO, is now available for user experiments. In this publication we will provide an overview of the TMO instrument including the various endstations, X-ray optics, optical laser systems, and available detectors. We conclude with a brief description of the main scientific research possible in TMO and some first results.

## 2. Instrument overview

**Figure 2:**
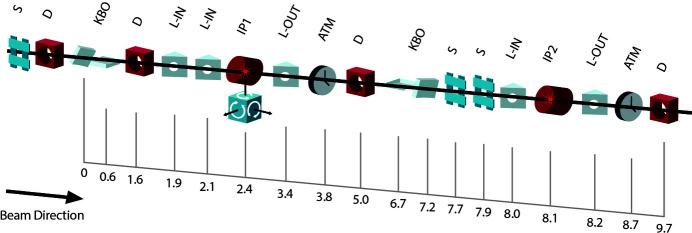
Schematic overview of the TMO instrument layout with distances. All distances are indicated in meters from the first mirror inside of TMO. Shown are scatter slits (S), diagnostics (D), KB optics (KBO), laser in-coupling (L-IN), interaction points (IP), laser out-coupling (L-OUT), and laser arrival time monitor (ATM).

To guarantee a high-flux soft-X-ray beamline, TMO stays in the NEH basement hutch formerly occupied by the atomic and molecular optics (AMO) instrument (see Fig. 1 ▸) (Bozek, 2009 ▸; Ferguson *et al.*, 2015 ▸) and has only one horizontal turning mirror between the undulators and the TMO instrument (see Fig. 4). All devices such as diagnostics, apertures, and optics are designed such that TMO can be operated in the energy ranges from 0.25 to 2.2 keV and 0.25 to 1.4 keV, and can take full advantage of both accelerators and the variable-gap soft X-ray undulators (Wallén *et al.*, 2016 ▸). The two different energy ranges result from the two different reflection cut-offs of the different mirror systems. The high-peak-power copper accelerator and the high-repetition-rate superconducting accelerator operate at 120 Hz and up to 1 MHz, respectively. The NEH 1.1 (TMO) instrument has two separate focus spots (*i.e.* interaction points) in series (see Figs. 2 ▸, 3 ▸, and 4 ▸). Each focus spot has a separate Kirkpatrick–Baez (KB) mirror (Baez & Kirkpatrick, 1946 ▸) system, focus diagnostic, sample delivery environment, laser module table, and controls system. Consequently, each focus spot can be operated independently but also simultaneously. The following subsection will give a more detailed description of TMO. An overview of the TMO instrument layout is given in Figs. 2 ▸ and 3 ▸.

### 2.1. X-ray optics

The essential X-ray focusing optics in TMO are KB mirrors. Each of the two KB systems are tailored to the applications of the corresponding focus spot, also called interaction points. Commonalities are the two different reflection zones, one B_4_C coated and one bare Si, to facilitate a maximum transmission over the energy range covered by TMO. The energy range of TMO is mostly defined by the material-dependent reflection cut-off with the given incident angle. The two different coatings of the mirrors can be reached with a translation of the mirror Si-substrate. Such a translation also allows for different reflection areas in case of mirror contamination. The incident angles with respect to the mirror surface are 14 mrad and 21 mrad for the first and second pair of KB mirrors, respectively. These incident angles allow for an X-ray energy range of 250–2200 eV on the first focus spot and an X-ray energy range of 250–1400 eV for the second focus spot. The first pair of mirrors are bendable in a plane-elliptical geometry which enables the foci to be varied dynamically along the first focus spot as well as to compensate for the aberration of the second pair of mirrors which are not bendable. The TMO focusing scheme is shown in Fig. 4 ▸. The two bendable mirrors are 600 mm long with 0.5 nm r.m.s. shape errors. The geometrical acceptance is larger than 2 FWHM for photon energies above 400 eV. They are equipped with benders for dynamic bending of the mirror with a reproducibility ten times better than what is needed to achieve the target focused spot size. The spot dimension can change from below 1 µm to about 200 µm FWHM. The minimum spot size depends on the photon energy for both interaction points (IPs). Fig. 5 ▸ depicts the focus parameters for the different photon beam energies. Details about the TMO KBO system are given by Seaberg *et al.* (2022 ▸).

### 2.2. Interaction point one (IP1)

At the first focus spot of the TMO instrument is the next-generation atomic, molecular, and accelerator science and technology experiments (NAMASTE) interaction point and it is optimized for performing high-energy, high-resolution, time-(but also angular)-resolved charged particle measurements. It accepts highly standardized modular endstations. The NAMASTE environment offers the possibility to install modular stations (roll in and out) which can be set up, aligned and commissioned outside the hutch and installed at the first TMO focus spot.

#### 2.2.1. LAMP

The LAMP instrument is a soft X-ray endstation for high-field physics and ultrafast science experiments. LAMP contains a high-resolution double-sided electron–ion coincidence velocity map imaging (VMI) spectrometer specifically designed for use in the LAMP end­station. This detects ions and/or electrons allowing for simultaneous and, under certain conditions, coincident measurements of all charged particles generated by photoionization of a single atom/molecule (Li *et al.*, 2021 ▸). More information on LAMP is given by Osipov *et al.* (2018 ▸).

#### 2.2.2. Magnetic bottle spectrometer

The TMO magnetic bottle electron spectrometer (MBES) features a 2.1 m flight tube and an electrostatic lens to retard high-energy photo-electrons, for improved kinetic energy resolution. The spectrometer has been integrated into the LAMP experimental chamber (Osipov *et al.*, 2018 ▸) but is designed to enable flexible integration with different endstations. An inhomogeneous magnetic field of strength 0.5 T is created at the interaction region by a neodymium permanent magnet mounted with a soft iron cone which acts as a magnetic shunt. A flat copper plate is attached to the nose cone, which enables the application of a uniform electric field across the interaction region by applying a voltage to the magnet. The tip of the iron cone sits 3 mm from the interaction region, opposite a copper nose cone also situated 3 mm from the interaction region. The aluminium flight tube has a 76 mm outer diameter and is mounted in vacuum. It is wrapped along its length by Kapton-insulated 22 AWG copper wire, through which a current of 1 A is flowed to create a uniform solenoid magnetic field of 1 mT. The electrons are detected by a micro-channel plate (MCP) detector coupled to an anode at the end of the flight tube. The detector is mounted opposite the flight tube in a four-way cross, with the remaining two ports occupied by an electrical feedthrough flange and a turbomolecular pump. The electrostatic lens consists of two pairs of plates and sits 19 cm from the interaction region. At this point, the magnetic field lines created by the combined fields of the permanent magnet and solenoid are almost fully parallel. To retard high kinetic energy electrons, the set of plates closest to the interaction region is held at ground while the other set of plates holds the retardation voltage. The flight tube is also held at the retardation voltage to ensure the electrons experience field-free drift after interacting with the electrostatic lens.

#### 2.2.3. Coaxial VMI

The coaxial VMI endstation provides an electron VMI spectrometer where the ionizing laser source propagates along the symmetry axis of the spectrometer. This coaxial VMI provides a unique two-dimensional projection of the three-dimensional electron momentum distribution. The coaxial imaging technique has been demonstrated in experiments at the Linac Coherent Light Source with both soft X-ray and infrared laser pulses. More information can be found in Li *et al.* (2018 ▸).

#### 2.2.4. Angular-resolved high-resolution electron spectrometer

Based on the design of the P04 beamline (Viefhaus *et al.*, 2013 ▸) at PETRA III diagnostic unit (so called ‘cookiebox’) (Lutman *et al.*, 2016 ▸; Hartmann *et al.*, 2018 ▸), TMO will feature an experimental and diagnostic endstation called MRCOFFEE (multi-resolution cookiebox optimized for the future of free-electron laser experiment) (Walter *et al.*, 2021 ▸). This multi-resolution spectrometer endstation is an angle-resolving array of 16 electron time-of-flight spectrometers that allow wide and adjustable energy acceptance windows (see Fig. 6 ▸). By interleaving detector retardations, it enables simultaneous angle-resolved photo-electron and Auger electron spectroscopy. The spectrometer array is available for: multiple-edge high-resolution photoelectron and Auger electron spectroscopy, spectral-polarimetry measurements as well as polarization-sensitive attosecond resolving temporal characterization of general LCLS-II pulses. This multi-polarization and multi-color spectral experimental endstation and/or diagnostic unit also has an energy resolution better than the expected seeding spectrum and the SASE spectral features.

#### 2.2.5. Future upgrades for IP1

Provisions are in place to accommodate additional experimental setups that would further increase the experimental capabilities at the TMO instrument. Possible future upgrades can be, but are not limited to, soft X-ray high-repetition-rate imaging and/or a photon spectrometer.

### 2.3. Interaction point two (IP2)

A new Dynamic REAction Microscope (DREAM) is positioned at the second interaction point. DREAM houses a well defined geometry and cold target recoil ion momentum spectroscopy (COLTRIMS) type spectrometer (Dörner *et al.*, 2000 ▸; Ullrich *et al.*, 2003 ▸) as a standard configuration to accommodate extreme vacuum, sub-micrometer focus spot size, and target purity requirements dictated by the pump–probe class of coincidence experiments, while accumulating data on an event-by-event basis at repetition rates in excess of 100 kHz, utilizing the LCLS-II capabilities (see Fig. 7 ▸). The photon-fluence in DREAM will reach over 10^21^ photons cm^−2^ in combination with the superconducting linac, while with the copper accelerator it will be over 10^22^ photons cm^−2^ at 120 Hz. The new DREAM instrument enables sophisticated coincidence measurement schemes for kinematically complete experiments at each time step of an evolving reaction. This experimental approach, known as a ‘molecular reaction microscope’, will enable the complete spatial reconstruction of the excited-state charge transfer and subsequent dissociation at each time step for a fixed-in-space molecular orientation. This is a powerful new approach for visualizing a broad range of excited-state molecular dynamics. With the high repetition rate of LCLS-II, with up to 1 MHz, it will push the boundaries of coincidence measurements (Kastirke *et al.*, 2020 ▸; Li *et al.*, 2021 ▸).

The standard detectors for this COLTRIMS endstation are two 120 mm state-of-the-art Hexanode delay-line detectors and a setup that allows tailored spectrometers sizes (length and diameter). A more detailed description of the DREAM endstation will be published elsewhere.

#### 2.3.1. Future upgrades for IP2

To overcome the limitations of a delay-line detector, SLAC is developing a spatial- and time-resolving front-end ASIC detector. This time-resolving pixelated detector is called TIXEL and would provide the time of arrival (TOA) and time over threshold (TOT). This new development is supposed to work without an MCP in front of the TIXEL detector. With such a pixelated charged particle detector based on silicon technology and with >300 hits at a repetition rate of up to 1 MHz it will be possible to perform for the first time massive coincidence experiments. Since the number of combinations grows exponentially with the number of hits for this detector it will be possible to detect and analyze large densities of hits with high repetition rate. Possible further future upgrades can be, but are not limited to, a compact jet system and/or aerosol environment. See Table 1 ▸ for the TIXEL parameters.

### 2.4. FEL capabilities

The soft X-ray line of LCLS-II is equipped with 21 variable-gap undulators. The soft X-ray (SXR) undulator can be operated with either the superconducting LCLS-II linac, or the normal conducting linac. In the first case the design energy range is between 250 eV and 2.5 keV (although the optics for the TMO hutch can only accept photon energies up to 2.2 keV), while in the second case the undulator can generate FEL radiation up to several keV.

The SXR line will leverage several advanced FEL capabilities developed with LCLS:

(i) Self-seeding will enable the generation of narrow-bandwidth radiation from the undulator (Ratner *et al.*, 2015 ▸).

(ii) XLEAP-II will generate attosecond pulses with tens of micro-joules of pulse energy and duration down to 200 as using enhanced SASE (Duris *et al.*, 2020 ▸; Zholents, 2005 ▸).

(iii) Two-color operation with the split undulator method will allow the generation of two pulses with wide energy separation (determined by the undulator tuning range) and delay control up to 1 ps (Lutman *et al.*, 2013 ▸).

Combining the XLEAP-II modulators with the two-color mode will enable X-ray pump/X-ray probe experiments with sub-femtosecond resolution. All three capabilities can be realized at high repetition rate using the superconducting LCLS-II linac.

Ongoing R&D aims at enabling femtosecond shaping of the electron beam to smoothly control the pulse duration (Marinelli *et al.*, 2016 ▸), advanced self-seeding to improve pulse-to-pulse stability (Lutman *et al.*, 2013 ▸) and double chirp/taper operation (Zhang *et al.*, 2019 ▸) for high-power two-color attosecond operation and single TW-scale pulses.

### 2.5. Optical laser capabilities

Optical excitations of samples in the target chambers are provided by synchronized, femtosecond laser pulses delivered from lasers that are situated in the NEH laser hall. Currently, these pulses are generated in Ti:sapphire chirped pulse amplification (CPA) systems, providing up to 20 mJ, ∼40 fs laser pulses at 800 nm and 120 Hz. Ultimately an in-house-developed optical parametric chirped pulse amplification (OPCPA) system will provide 800 nm, 100 kHz, 35 W, <20 fs pulses (Mecseki *et al.*, 2019 ▸). Each endstation has an associated laser delivery setup, consisting of a series of modules for conditioning and manipulating the drive laser, with the possibility of generating pulses ranging from UV wavelengths to THz frequencies. An emphasis has been placed on the incorporation of numerous control and diagnostic points throughout the laser delivery system, to monitor the system performance, improve reliability, and allow for greater remote control of laser properties. Laser in-coupling is tailored to the specific needs at each interaction point. For example, in-coupling to the DREAM endstation is designed to produce a <10 µm FWHM focal spot, whereas two in-coupling paths are incorporated into IP1, one for UV–visible wavelengths and a separate path for near-infrared (NIR)–mid-infrared (MIR) wavelengths. In order to determine a shot-by-shot time-of-arrival of the laser pulse relative to the X-rays, a small portion of the laser pulse is used to perform a cross-correlation with the X-rays (Muhammad *et al.*, 2021 ▸). This enables re-sorting of data into appropriate time bins, improving the temporal resolution of measurements. The incorporation of a distributed, laser-based timing system into these cross-correlation measurements will provide a reference clock that is accurate to the ∼1 fs level, and is expected to enable temporal resolution below 10 fs.

### 2.6. Sample delivery

The new TMO instrument provides several different gas phase and aerosol delivery options as well as the option to host condensed and solid phase targets. A new remote-controllable and automated sample delivery system is part of TMO. Each interaction point has its own gas delivery environment, roughing line system and exhaust lines to make sure both interaction points can be operated completely independent. TMO can provide an Even–Lavie valve (Even, 2015 ▸), high-pressure Parker valve, a tailored CW jet, aerosols and effusive sample gas delivery systems, flow cell, as well as sample stages for solid targets. As a future upgrade, a high-repetition-rate pulsed gas jet is foreseen (<5 kHz). To keep the high transmission of the mirror system and avoid contamination, liquid jets are not foreseen at TMO.

### 2.7. Diagnostics

**Figure 3:**
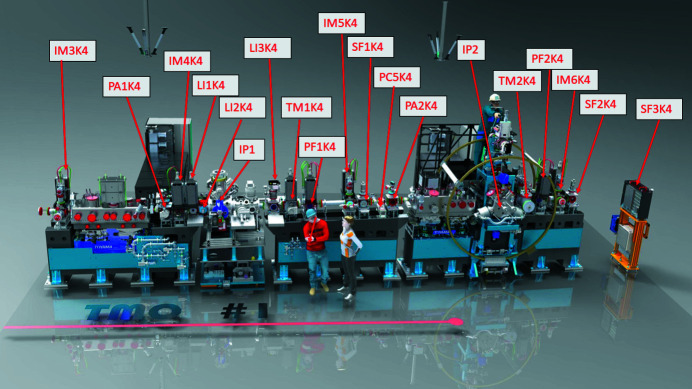
Overview of the TMO instrument layout, showing both endstations which are capable of taking full advantage of both the high per-pulse energy from the copper accelerator (120 Hz) as well as high average intensity and high repetition rate from the superconducting accelerator (1 MHz). Indicated are the beam-position monitors IM3K4, IM4K4, IM5K4, IM6K4; differential pumping sections PA1K4 and PA2K4; IP1 optical laser in-coupling LI1K4, LI2K4, and IP1 laser out-coupling LI3K4; the arrival-time monitors TM1K4 and TM2K4; wavefront sensor PF1K4 and PF2K4; retractable beam terminators SF1K4 and SF2K4; beamline collimators PC5K4; as well as the interaction points IP1 and IP2; and finally the beam terminator SF3K4.

**Figure 4:**
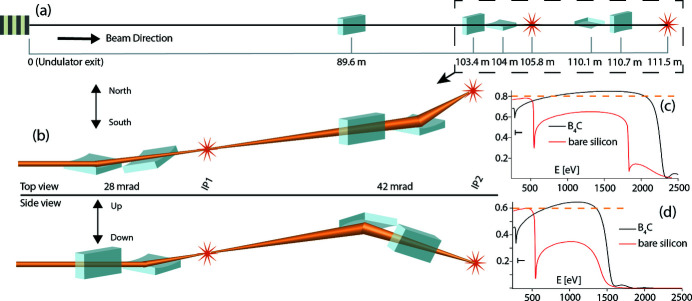
Overview of the TMO mirror layout. (*a*) The distances of each mirror and IP from the undulator in meters. The shown section inside the dashed line marks the TMO hutch where the KBO systems reside. (*b*) The dashed line section from (*a*) with the mirror orientations and deflection angle as top and side view. (*c*) The transmission (*T*) over the photon energy (*E*) for IP1 for both mirror coatings. (*d*) The transmission (*T*) over the photon energy (*E*) for IP2 also for both mirror coatings. A horizontal dashed line indicates the design goal for the reflectivity after all the mirrors; it is 80% for IP1 and 60% for IP2.

The TMO instrument hosts a suite of different destructive and non-destructive diagnostics to characterize the X-rays. Beside standard beam-positioning monitors along the beam trajectory (see Fig. 3 ▸) each focus spot in TMO has its own wavefront sensor (WFS) using the fractional Talbot effect (Liu *et al.*, 2020 ▸). Given the high repetition rate of the FEL each WFS will be operated in an average mode. Each WFS, however, can be used with the full beam transmission. The destructive nature of this kind of measurement makes the simultaneous use of both focus spots with both WFSs in­accessible. To measure the beam transmission downstream of every mirror system, TMO has power meters after each X-ray optic (Heimann *et al.*, 2019*b* ▸). To measure the position of the X-ray beam on each mirror and for non-invasive, pulse-by-pulse normalization a fluorescence intensity monitor (FIM) has been developed (Heimann *et al.*, 2019*a* ▸) and will be available as a standard TMO diagnostic on each TMO mirror. A photon spectrometer based on an off-axis Fresnel zone plate optic is currently under development. This device will serve as a shot-to-shot diagnostic of the X-ray spectrum. The spectrometer will be deployed between IP1 and IP2. The final design and performance will be published elsewhere. To measure the average, absolute, and pulse-resolved photon flux of the FEL beam, two gas monitor detectors (GMDs) are implemented before and after the gas attenuator (Tiedtke *et al.*, 2008 ▸, 2014 ▸). The gas attenuator is located in the front-end enclosure (FEE) shortly after the undulator section and before the TMO hutch. Table 2 ▸ gives an overview of the TMO parameters and capabilities.

## 3. TMO science

With the focus of TMO on ultrafast X-ray atomic and molecular physics, we have a powerful tool to tackle new challenges: charge migration, redistribution, and localization as well as symmetry break down and chirality, even in simple molecules, are not well understood at the quantum level. These fundamental phenomena are central to complex processes such as photosynthesis, catalysis, and bond formation/dissolution that govern all chemical and biochemical reactions. Ultrafast soft X-rays at high repetition rate from LCLS-II will provide qualitatively new probes of excited-state energy and charge flow and how they work in simple and complex molecular systems. The following section will give an exemplary overview of science cases enhanced by TMO and LCLS-II.

### 3.1. Investigation of ultrafast processes with attosecond soft X-ray laser pulses

As attosecond science plays a central part in our LCLS-II scientific mission, TMO gives the possibility to follow fundamental phenomena from attosecond charge migration involving electronic dynamics and correlation to femtosecond charge transfer involving nuclear rearrangement. Understanding charge dynamics at the microscopic level is essential not only to achieving fundamental insights into these phenomena but also, on a longer-term perspective, for improving applications relying on charge motion and separation, from artificial photosynthesis and molecular electronics to photocatalytic and photovoltaic devices. The continuous tunability and orders-of-magnitude pulse energy increase (compared with any previous attosecond source) produced by enhanced SASE operation at the LCLS enable a suite of nonlinear spectroscopies which are, at the moment, unavailable elsewhere. Therefore, the new capabilities of TMO and LCLS-II can be used to perform measurements demonstrating the control and observation of coherent electronic motion on its natural attosecond timescale, and explore how it affects the subsequent motion of the nuclei to drive chemical change (Li *et al.*, 2022 ▸).

### 3.2. Ultrafast dynamics in chiral molecules

With the new features of LCLS-II, *i.e.* a polarization-tunable undulator, sub-femtosecond pulses, multibunch modes, up to 1 MHz repetition rate (see Section 2.4), as well as the new TMO instrument with the above-described capabilities, SLAC has opened the door for unprecedentedly deep insights into chirality. It will finally be possible to observe how chiral systems form, restructure, and how their functionality can be understood or even controlled. Investigating the origin of chirality with TMO will build bridges between the macro-biological level, chemical dynamics and the fundamental spin properties of matter (Pitzer, 2017 ▸; Hartmann *et al.*, 2016 ▸; Ilchen *et al.*, 2021 ▸).

### 3.3. Ultrafast photochemistry

In combination with a high-repetition-rate soft X-ray source, the TMO hutch is well equipped for the investigation of ultrafast photochemistry. The high average X-ray flux will allow the measurement of orders-of-magnitude weaker signals using already established time-resolved experimental methods (Wolf *et al.*, 2017 ▸; McFarland *et al.*, 2014 ▸). Furthermore, the high repetition rate will enable novel experimental techniques based on coincidence detection, allowing for example to associate spectral signatures with specific fragmentation patterns. Such techniques will help in differentiating multiple parallel photochemical reaction channels and in detecting reactive processes with small quantum yields, which can nevertheless be of significant interest, *e.g.* for organic chemistry.

### 3.4. Future flagship capabilities

With the new TMO beamline and the two X-ray focus spots in line, TMO will be able to characterize the substructure of SASE pulses and correlate it with the time-resolved study of light–matter interactions for charged particle spectroscopy. It has been shown that the temporal sequence of spikes can crucially influence the underlying ionization pathways (Li *et al.*, 2021 ▸). Moreover, at very short pulse durations, SASE pulses are spectrally broad and therefore each pulse possesses a specific temporal and spectral profile, which ultimately determines the non-linear excitation process in all FEL-related photon energy regimes. For the SASE pulse characterization, we plan to implement the method of ‘angular streaking’ at TMO IP1 with MRCOFFEE (Hartmann *et al.*, 2018 ▸; Walter *et al.*, 2021 ▸). Enabled by this novel technique, we will spectrally and temporally characterize the SASE pulses at the IP1, including their single-shot duration with sub-femtosecond resolution, with the full time–energy information for each incoming XFEL shot (Driver *et al.*, 2020 ▸). The DREAM endstation in IP2 will be a superior endstation to perform linear-, high field-, coincidence-, covariance-, and Coulomb explosion imaging-experiments with full analysis of the X-ray pulse spectrum. It will lead to a complete analysis of the underlying electron dynamics from the birth of a (photo)-electron to the early state of charge migration, charge transfer, to nuclear motion and chemical reactions.

## 4. TMO first results

**Figure 5:**
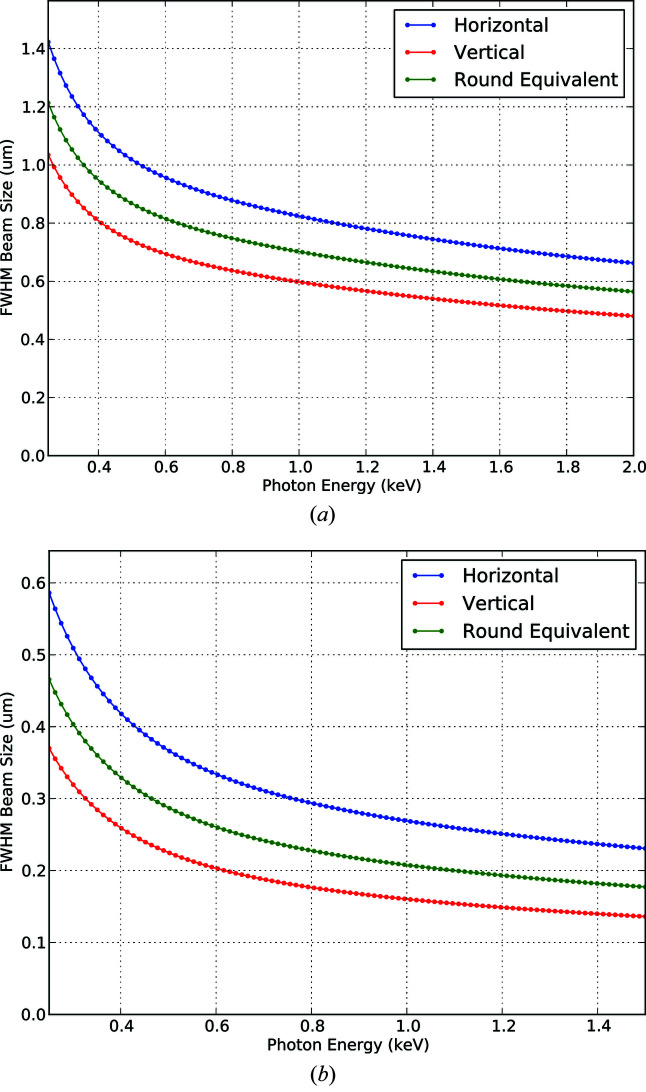
Exemplary smallest achievable focus spot sizes at both IPs in TMO, obtained from calculations based on start-to-end simulations for LCLS-II with 20 pC bunch charge. The top panel refers to the IP1 (NAMASTE) reaching 2 keV. The bottom panel refers to the IP2 (DREAM) chamber and can reach 1.4 keV.

**Figure 6:**
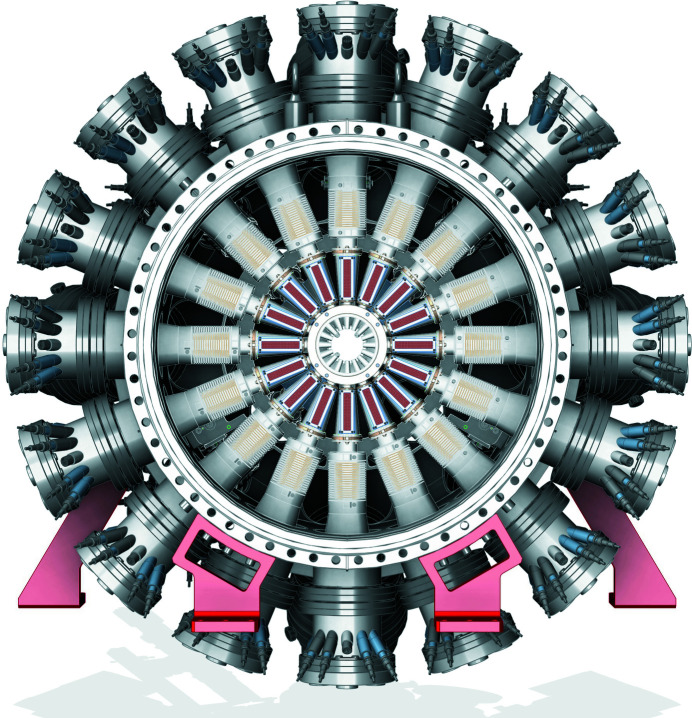
Axial view of the multi-resolution electron spectrometer array detector along the direction of X-ray propagation, showing the 16 azimuthal eToFs MRCOFFEE image taken from Walter *et al.* (2021 ▸).

**Figure 7:**
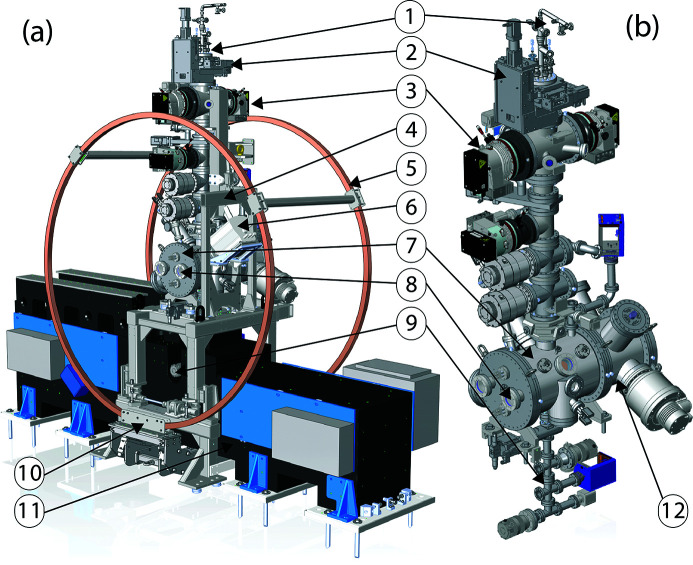
Overview of the DREAM instrument shown as endstation mounted on the support granite (*a*), and the chamber assembly without support infrastructure (*b*). Indicated are the CW supersonic jet (1), the three-axis jet manipulator (2), the first skimmer stage pumping (3), the four skimmer section (4), the Helmholtz coil pair (5), the long-range microscope (6), the main chamber (7), the close optical laser in-coupling next to the out-coupling (8), the two-stage catcher section with residual gas analyser (RGA) (9), the three-axis coil mover (10), the support granite (11), and the extension chamber (12).

**Figure 8:**
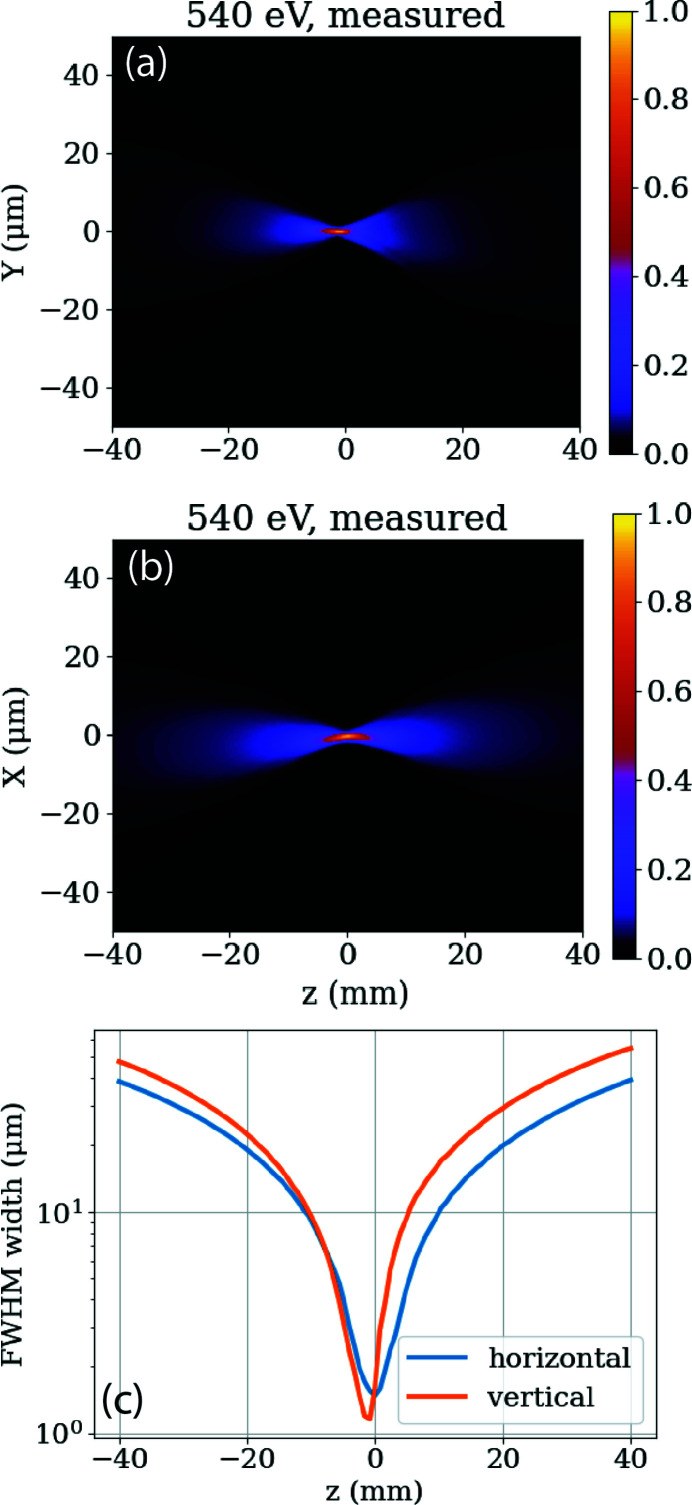
First wavefront measurements at the IP1 focus spot of TMO. Shown are the horizontal measured line-outs along the beam (*a*), the vertical measured line-outs along the beam (*b*), as well as the FWHM for both (*c*).

The TMO hutch opened the X-ray stoppers for the first photons on 9 October 2020 and had its first successful experiment only one month later. At first, TMO conducted a series of high-field experiments to demonstrate its main new capabilities. Most of the results of this first series of experiments will be published elsewhere. Here we present some selected results. The first wavefront measurement was made during the first week of commissioning. Fig. 8 ▸ shows the line-outs of the horizontal and vertical focus, as well as the FWHM for both focus orientations. The residual astigmatism and some minor coma are expected to be removed during future mirror bender optimizations. The horizontal and vertical outlines of about 1 µm are in good agreement with the calculated values shown in Fig. 5 ▸. A future publication to show the performance of the wavefront sensor validated via imprints is underway and will be published elsewhere.

**Figure 9:**
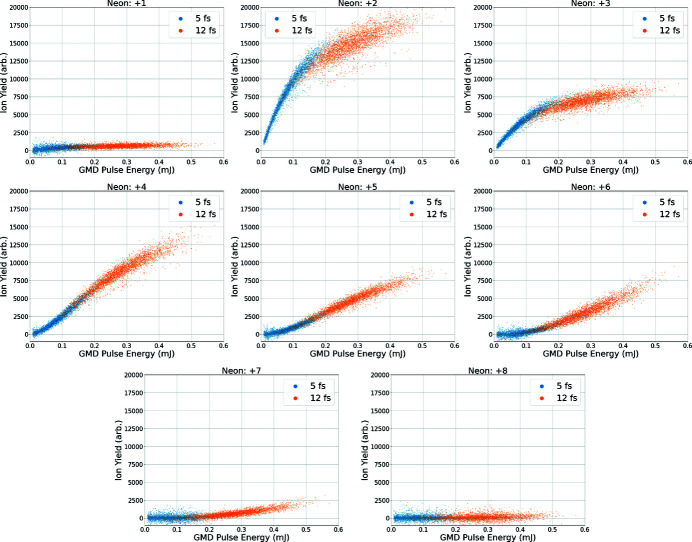
Ratio of charge state yield versus XGMD pulse energy for 5 fs (blue) and 12 fs (orange) electron bunch duration and at a photon energy of 1050 eV. Pulse energies are measured in the XGMD upstream of the target. The charge states 9+ and 10+ were not observed in the single-shot spectra. The shown ratios are in good agreement with the literature for this photon energy (Young *et al.*, 2010 ▸).

To demonstrate the high intensities possible at the first interaction point of TMO, we investigate the sequential ionization of neon atoms at 1050 eV, originally considered by Young *et al.* (2010 ▸). Fig. 9 ▸ shows the yield of various charge states as a function of X-ray pulse energy, measured using the X-ray gas monitor detector (XGMD), collected by the velocity map imaging spectrometer in LAMP. This figure displays data taken with two different pulse durations, 5 fs and 12 fs (FWHM), produced using the slotted foil technique (Emma *et al.*, 2004 ▸; Ding *et al.*, 2015 ▸). At 1050 eV we expect the ionization dynamics to be dominated by the sequential core-level photoionization-Auger (PA) process, although other ionzation mechanisms (valence and inner-valence ionization, V) are also possible. The PA process will proceed until we reach the ground state of berillium-like neon (Ne^6+^), where the binding energy of the core-level electrons is greater than the incident photon energy. Continued ionization of valence electrons will lead to helium-like neon (Ne^8+^). We found no significant yield of charge states beyond Ne^8+^, and observe a clear saturation effect for the Ne^2+^ fragment. While it is difficult to compare with the previous measurement since the VMI spectrometer is an open area detector and Young *et al.* employed a spatially resolving detector, the degree of ionization of the target seems to be reduced in the current data set. This observation along with the clear saturation effect in the lower charge state points to the X-ray induced transparency effect observed by Young *et al.* at higher photon energies, although further modeling of the ionization process is needed to fully confirm this.

**Figure 10:**
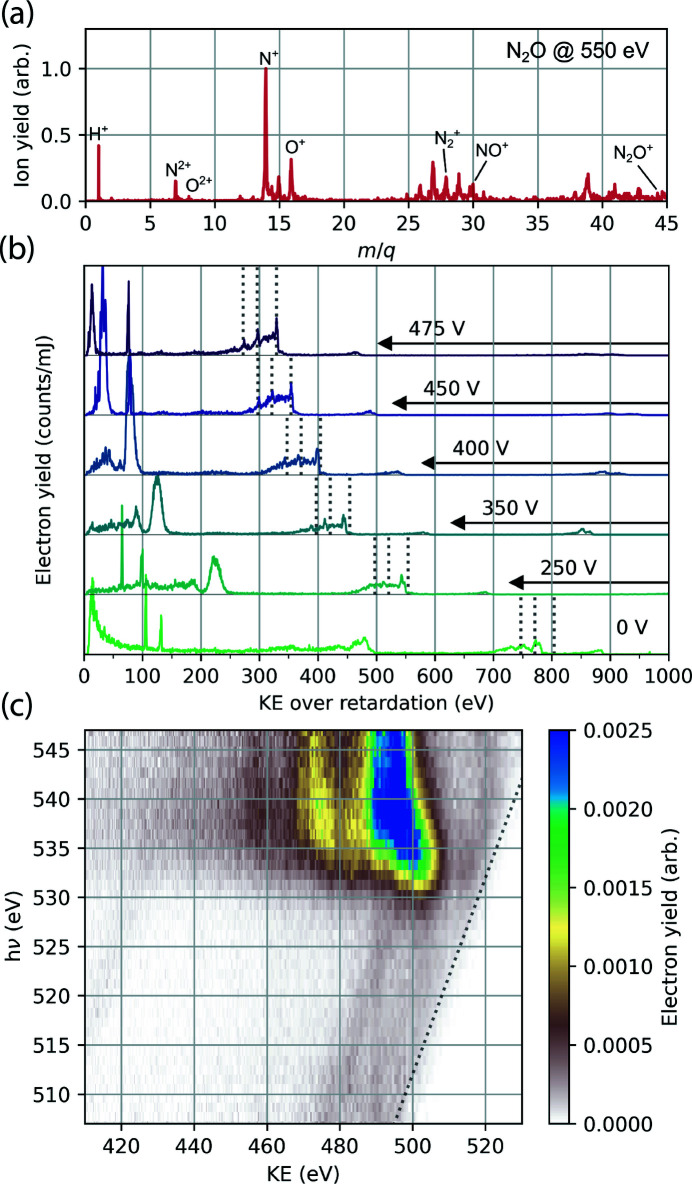
(*a*) Ion mass-to-charge spectrum of N_2_O irradiated by 550 eV X-ray pulses recorded with the magnetic bottle spectrometer. (*b*) Photoelectrons produced by ionization of Ne atoms by 1.35 keV X-rays, recorded at different retardation voltages applied to the electrostatic lens. As the retardation voltage is increased, the manifold of three primary peaks which form the Ne *KLL* Auger–Meitner emission spectrum becomes more sharply defined and aligns with previously measured values, as shown by dotted gray lines (Krause *et al.*, 1970 ▸). (*c*) Resonant Auger–Meitner electron emission map of gas-phase 4-aminophenol, recorded with the MBES. Central photon energy of incoming X-ray pulse is plotted on the *y*-axis and electron kinetic energy is plotted on the *x*-axis. At photon energies corresponding to the manifold of O 1*s* → valence excitations in the molecule (535 eV) there is a resonant enhancement in the Auger–Meitner electron yield. The emission converges to normal *KLL* Auger–Meitner decay above the O *K*-edge at 540 eV. The highest kinetic energy photoelectrons are produced by X-ray ionization of the valence shell and show the expected linear dispersion with photon energy (marked by the dotted line).

Further results are from the MBES. Fig. 10 ▸ illustrates the performance of the TMO MBES. The top panel shows the ion mass-to-charge spectrum recorded following X-ray ionization of nitrous oxide at a photon energy of 550 eV. To collect ions, a voltage of +5 kV is applied to the copper plate mounted to the permanent magnet and the lens retardation voltage is held at 0 V. The middle panel shows the electron kinetic energy spectrum of photoemission from neon gas at an X-ray photon energy of 1.35 keV, with different retardation voltages applied to the electrostatic lens. The retardation voltage is shown on the right-hand side of the figure. Increasing the retardation voltage slows the photoelectrons and improves kinetic energy resolution. The manifold of peaks at 800 eV kinetic energy corresponds to Ne *KLL* Auger–Meitner emission, which is dominated by three primary features (marked with dashed lines in Fig. 10 ▸) each separated by 30 eV (Krause *et al.*, 1970 ▸). At higher retardation voltages, these three peaks become clearly resolved. The magnetic bottle spectrometer is an ideal detector for resonant Auger–Meitner (AM) spectroscopy as demonstrated in Fig. 10 ▸(*c*). Gas-phase 4-aminophenol sample is ionized by X-ray pulses from the tunable undulators. We scan the photon energy across the near-edge absorption features near the oxygen *K*-edge and record the spectrum of the photoemission products with 400 V on the retardation plates. Between 530 and 535 eV incident X-ray energy we observe the resonant AM spectrum. It is difficult to resolve any features in the AM spectrum, which is likely a consequence of the density of cationic states accessible through the AM process. Above 538 eV the AM spectrum shifts to lower kinetic energy indicating a transition to the normal AM process where *K*-shell electrons are ionized rather than excited.

## 5. Conclusion

LCLS and LCLS-II produce high-flux few to sub-femtosecond X-ray pulses, yielding unprecedented X-ray intensities. The TMO instrument takes advantage of the pulse properties to perform high-power soft X-ray experiments in a wide spectrum of scientific domains. The instrument provides users with a variety of endstations, spectrometers, and other components for the utmost flexibility in experimental layouts and signal detection schemes. The latest updates and more details about the TMO instrument can be found on the following website: https://lcls.slac.stanford.edu/instruments/neh-1-1.

## 6. Facility access

LCLS instruments are open to academia, industry, government agencies, and research institutes worldwide for scientific investigations. There are two calls for proposals per year and an external peer-review committee evaluates proposals based on scientific merit and instrument suitability. Access is without charge for users who intend to publish their results. Prospective users are encouraged to contact instrument staff members to learn more about the science and capabilities of the facility, and opportunities for collaboration.

## Figures and Tables

**Table 1:** TIXEL key performance parameters

Mode of operation	TOT and TOA
Time resolution	<100 ps
Pixel size	100 µm × 100 µm
Energy range	1–10 keV
Array	176 × 192
Si technology	0.13 µm
Full matrix readout	5 kfps

**Table 2:** Parameters and capabilities of the TMO instrument

TMO parameter	IP1	IP2
Energy range (eV)	250–2200	250–1400
Mirror incidence angle (mrad)	14	21
Mirror coating	Si or B_4_C	
Smallest focus (µm)	<1.5	<0.3
Flux, up to (photons cm^−2^)	10^21^	10^22^
Mirror transmission (%)	80	60
Pulse duration (fs)	0.3–100	
Repetition rate X-rays (kHz)	Up to 929	
Polarization control	H, V, and elliptical/circular R, L	
Target sample	Gas jet, aerosol, solid	
Gas sample delivery	CW and pulsed valves	
Gas sample delivery temperature (K)	10–650	
Optical laser source	OPCPA	
Laser peak intensity on sample (W cm^−2^)†	10^14^	10^14^
Repetition rate OPCPA (kHz)‡	100	
Optical laser wavelength (µm)	0.2–10	0.2–5
Optical laser focus spot size (µm)§	25	<10

† At 100 kHz and 800 nm.

‡ Later upgrade to MHz.

§ At 800 nm.
