# The time-resolved atomic, molecular and optical science instrument at the Linac Coherent Light Source. Corrigendum

**DOI:** 10.1107/S1600577525005715

**Published:** 2025-06-26

**Authors:** Peter Walter, Timur Osipov, Ming-Fu Lin, James Cryan, Taran Driver, Andrei Kamalov, Agostino Marinelli, Joe Robinson, Matthew H. Seaberg, Thomas J. A. Wolf, Jeff Aldrich, Nolan Brown, Elio G. Champenois, Xinxin Cheng, Daniele Cocco, Alan Conder, Ivan Curiel, Adam Egger, James M. Glownia, Philip Heimann, Michael Holmes, Tyler Johnson, Lance Lee, Xiang Li, Stefan Moeller, Daniel S. Morton, May Ling Ng, Kayla Ninh, Jordan T. O’Neal, Razib Obaid, Allen Pai, William Schlotter, Jackson Sheppard, Niranjan Shivaram, Peter Stefan, Xiong Van, Anna Li Wang, Hengzi Wang, Jing Yin, Sameen Yunus, David Fritz, Justin James, Jean-Charles Castagna

**Affiliations:** a SLAC National Accelerator Laboratory 2575 Sand Hill Road Menlo Park CA94025 USA; b Stanford PULSE Institute SLAC National Accelerator Laboratory 2575 Sand Hill Road Menlo Park CA94025 USA

**Keywords:** LCLS-II, FEL, AMO, attosecond

## Abstract

The name of an author in the article by Walter *et al.* (2022) [*J. Synchrotron Rad.***29**, 957–968] is corrected.

## Abstract 2

Corrigendum to the article by Walter *et al.* (2022) [*J. Synchrotron Rad.***29**, 957–968].

The name of one of the authors in the article by Walter *et al.* (2022 ▸) is incorrectly given as Jackson Shepard. The correct spelling is Jackson Sheppard, as given above.
